# Which is the IDEAL Peginterferon for Hepatitis C: A Meta-Analysis of both Pegylated Interferons in the Treatment of HCV-Infected Patients

**Published:** 2010-09-01

**Authors:** Frank Tacke

**Affiliations:** 1 Department of Medicine III,RWTH-University Hospital Aachen, Aachen, Germany

Chronic infections with the hepatitis C virus (HCV) represent a major global health problem, with around 170 million patients at risk of developing life-threatening complications such as liver cirrhosis or hepatocellular carcinoma. The standard treatment of care is a combination of weekly pegylated interferon (peginterferon) alfa and daily ribavirin for 24-72 weeks, dependent on HCV genotype and the patient's individual virological response to therapy [1][2]. Currently, there are two forms of peginterferon licensed for the treatment of hepatitis C: peginterferon alfa-2a (Pegasys, Roche) and peginterferon alfa-2b (Pegintron, Schering-Plough). Although direct pharmacodynamic comparisons of both substances have shown that they differ considerably with respect to pharmocokinetics and initial virological suppression [3], it remained largely unclear whether this is relevant for clinical endpoints, especially for achieving sustained virological response in HCV-infected patients

In fact, the largest phase-3 trials for peginterferon alfa-2a and alfa-2b initially yielded somewhat similar outcomes [4][5], establishing a "gut feeling" among many hepatologists worldwide that both substances were very likely equally potent in clinical practice. This was strongly supported by the largest endpoint trial to directly compare these two peginterferons, the IDEAL trial. In this trial, more than 3,000 patients were randomized to receive either peginterferon alfa-2b (at two different doses, i.e. 1.0 or 1.5 µg/kg per week) or alfa-2a (at 180 µg/week), and similar sustained virological response (SVR) rates were reported for all three treatment groups [6]. Two smaller recent studies reported by Rumi and Ascione this year[7][8] challenged this conclusion by revealing a modest but significant increase in SVR for patients treated with peginterferon alfa-2a(Table 1).

available high-quality studies. Prof. Alavian and colleagues present in this issue of Hepatitis Monthly a thorough, up-to-date, and extensive meta-analysis of studies comparing the efficacy of the two peginterferons in patients chronically infected by HCV [9]. This meta-analysis included 3,518 patients from 7 randomized controlled trials and revealed that peginterferon alfa-2a is superior to alfa-2b with respect to efficacy, namely the early effects, end-of-treatment outcomes, and sustained virological response. Although a higher rate of neutropenia was observed in the alfa-2a treated patients, the overall safety profile of both drugs and withdrawal rates were similar [9]. This interesting study by Alavian and colleagues is in full agreement with an independent meta-analysis performed by a group of researchers from Copenhagen [10], which also reported higher SVR in patients treated with peginterferon alfa-2a than in patients treated with alfa-2b.

What implications do these results have for clinical practice? In my opinion, collectively, these studies strongly indicate that peginterferon alfa-2a plus ribavirin should be the first choice of treatment for treatment-naïve, HCV-infected individuals. This is likely most relevant for genotype-1-infected cases, as most data in the existing literature were obtained from these patients. However, this might not hold true for all patients. For instance, patients with low levels of pretreatment white blood cells might potentially benefit more from peginterferon alfa-2b (or at least be at lower risk of harm). Furthermore, for patients in retreatment after HCV relapse, more data are currently available for the use of peginterferon alfa-2b [11]. Undoubtedly, both peginterferons are efficient drugs and similarly safe; the hepatologist's ultimate decision to use one or the other peginterferon should take into account not only the efficacy data from Alavian's meta-analysis, but also the individual patient's characteristics, comorbidities, compliance issues, local experiences, and economical considerations.

**Table 1:** Comparison of efficacy data from three recent, direct-comparison trials of peginterferon alfa-2a plus ribavirin and peginterferon alfa-2b plus ribavirin in the treatment of (naive) HCV-infected individuals.

**Prinicipal investigator**	**HCV genotype­**	**Patient Numbers**	**EoT**		**SVR**	
			**alfa-2a**	**alfa-2b**	**alfa-2a**	**alfa-2b**
McHutchison [6]	1	3070	64.4%^a^	53.2%	40.9%	39.8%
Rumi [7]	1, 2, 3, 4	431	78%^a^	67%	66%^a^	54%
Ascione [8]	1, 2, 3, 4	320	83.8%^a^	64.4%	68.8%^a^	54.4%

^a^ EoT: end of treatment response; SVR: sustained virological response;difference was statistically significant(P < 0.05)
